# Differences between medically treated and untreated non-fatal self-harm reported by hotline callers in China

**DOI:** 10.7717/peerj.7868

**Published:** 2019-10-17

**Authors:** Yongsheng Tong, Yi Yin, Nancy H. Liu

**Affiliations:** 1 Beijing Suicide Research and Prevention Center, Beijing Huilongguan Hospital, Beijing, China; 2 WHO Collaborating Center for Research and Training in Suicide Prevention, Beijing, China; 3 Peking University Huilongguan Clinical Medical School, Beijing, China; 4 Department of Psychology, University of California, Berkeley, Berkely, CA, USA

**Keywords:** Self-harm, Medical treatment, Hotline, Suicide intention

## Abstract

**Background:**

Many self-harmers do not present in hospitals due to the self-harm. It is still unclear on the differences between medically treated and untreated self-harm in China. This study described the differences of the two groups of self-harmers using the largest psychological aid hotline data.

**Methods:**

The present observational study recruited 3,403 hotline callers who reported episodes of self-harm before the call. In routine assessment, information about the most recent episode of self-harm was collected, including the method of self-harm, the wish to die, goals of the self-harm, and any medical treatment (irrespective of psychological services) in the hospital. The callers were divided into two groups: those who received hospital-based medical treatment due to the most recent self-harm (treated self-harm callers) and those who did not (untreated self-harm callers).

**Results:**

In the most recent episode of self-harm, 65% (*n* = 2,217) of callers were untreated and 55% (1,226/2,217) of the untreated self-harm callers reported a wish to die. A total of 67% of the callers reported that their main goal of self-harm was to relieve suffering. The most common self-harm methods were using instruments (knife or rope) and overdosing on medicines. Compared with treated self-harm callers, the untreated self-harm callers were less likely to have a wish to die (OR = 0.57), engage in self-harm outside the home (OR = 0.71 and 0.78), and attribute their self-harm to romantic relationship problems (OR = 0.76); however, they were more likely to use instruments, to jump, or to choose other methods (OR = 3.73, 3.83, and 7.71, respectively).

**Conclusions:**

Among hotline callers, many episodes of self-harm did not receive medical treatment, despite over half reporting a wish to die. Characteristics of self-harm behaviors were different between treated and untreated self-harm callers. Our findings suggest that more strategies should improve access to hospital-based medical treatment and coverage for post-intervention for self-harmers who are not presented in hospitals.

## Introduction

Self-harm, which refers to intentional bodily self-harm irrespective of motivation and degree of suicide intent, is a global public health problem (Haagsma et al., 2016). Non-fatal self-harm is a strong predictor of the subsequent suicide (Beautrais, 2004; Runeson et al., 2016) or repeated self-harm (Murphy et al., 2012; Kwok et al., 2014). Despite the mounting literature on self-harm or suicidal behavior, it is still unclear whether characteristics of the specific self-harm behaviors among those who received medical treatment are different from those who did not. This issue has been partially addressed by describing these two groups separately or are otherwise based on convenient samples drawn from hospital settings (Beautrais, 2004; Cooper et al., 2010; Murphy et al., 2012; Caterino et al., 2013; Haagsma et al., 2016; Tong et al., 2013a; Xu et al., 2013; Kwok et al., 2014; Lyons et al., 2016; Runeson et al., 2016). However, the majority of individuals with self-harm did not present to hospitals nor were registered in the self-harm surveillance system (Geulayov et al., 2018). Therefore, the characteristics of individuals presenting with non-fatal self-harm who did not receive prior treatment have not been fully investigated and understood. Some studies reported on the self-harm of adolescents or college students in communities, middle/high schools, or universities and colleges; however, their treatment-seeking behaviors were not compared (O’Connor, Rasmussen & Hawton, 2012; Law & Shek, 2013; Liu et al., 2018; Tang et al., 2018). Several studies have described characteristics of self-harm among those who sought for medical or other professional help (Milner & De Leo, 2010). Considering the large proportion of untreated acts among self-harm (Geulayov et al., 2018), it is important to explore and understand the characteristics of individuals presenting with self-harm who did not receive hospital-based medical treatment. Such an understanding would contribute to developing more specific and effective prevention strategies for the self-harm, and, to some extent, help us estimate the prevalence of non-fatal self-harm in China more accurately.

Hotlines (or crisis lines, lifelines) deliver various psychological service to suicidal individuals (Gould et al., 2012; Tan et al., 2012). They also provide a good opportunity for collecting information from callers with a history of non-fatal self-harm, regardless of having received medical treatment or not. The Beijing Psychological Aid Hotline was established in 2002 and has received more than 200,000 nation-wide calls. Half of the callers reported suicidal ideation and nearly 18% reported a history of self-harm (Tong et al., 2013b). In this study, we compared untreated self-harm callers (i.e., who did not receive hospital-based medical treatment (irrespective psychological services) due to the most recent episode of self-harm) with treated self-harm callers (who received such treatment due to the most recent episode of self-harm), as well as their methods, reasons, goals of self-harm behaviors, and wish to die, etc. Given that seeking treatment may be associated with the severity of self-harm, and lethality of methods may be related to the wish to die, we postulated that the methods of self-harm and wish to die would distinguish the untreated self-harm callers from the treated self-harm callers.

## Materials and Methods

### Setting and participants

The present study was an observational study. The data on episodes of self-harm were collected from the Beijing Psychological Aid Hotline in China. The hotline services was for all Chinese; only a quarter of the callers lived in Beijing. Nearly half of the callers sought help due to psychological problems. During a standard call, trained hotline operators routinely assessed relevant information about ongoing self-harm act (if had) or past self-harm history, and demographic characteristics of the caller. A structured form would appear automatically on the screen to help the operator to collect, code, and record data accurately. This process took less than 10 min and was an important component of the hotline service. The phone number of the incoming call was used to identify whether it was a repeat call from a previous caller. If a caller reported that he or she had used another phone number to call the hotline service previously, then calls from previous numbers would be linked with the current number.

In the present study, only calls from callers seeking psychological help for themselves were eligible. As we were interested in previous treatment and self-harm behaviors, exclusion criteria were as follows: (a) those reporting no history of self-harm behavior prior to this call; (b) a prior self-harm behavior was reported, but no data was available on whether the caller had been medically treated in hospital (irrespective of psychological services) due to the reported self-harm episode; or (c) repeated calls from the same caller. For the repeated calls from the same caller, only the call with the most coded variables about self-harm was selected. If the number of coded variables was the same among several repeated calls of the same caller, the earliest call in which reported an episode of self-harm would be selected.

The study was approved by the Institutional Review Board of Beijing Huilongguan Hospital (2014-106). The study conformed to the Declaration of Helsinki. Each caller was informed by a voice message that related data would be collected and analyzed anonymously before the hotline being connected. All information regarding the identity of the hotline callers was anonymized and de-identified before analyses.

### Measurements

In the present study, non-fatal self-harm refers to intentional bodily self-harm without an outcome of death (Haagsma et al., 2016). Given the time restriction with hotline calls, for each call, only eight questions on non-fatal self-harm acts were asked in the routine hotline assessment, which were as follows: (1) how many episodes of self-harm has the caller had? (2) When did the most recent episode of self-harm occur? Based on the most recent episode, six multiple choice questions would be asked, which included: (3) Did the caller have a wish to die by the act at the time self-harm occurred (yes or no); (4) Whether the caller had received inpatient or outpatient hospital-based medical treatment (yes or no, irrespective of psychological services) due to the self-harm, regardless of the details of medical treatment; (5) Where did the episode of self-harm occur (at home, workplace, or other public places); (6) What method of self-harm did the caller use for the most recent episode (medication overdose, other poisons, instrument such as knife etc., jumping, or other methods); (7) What did the caller consider as the main reason for the self-harm behavior (problems in romantic relationship, family conflicts, conflicts with persons outside family, work or study problems, depression, etc.); and (8) What was the main goal of the self-harm (decreasing others’ burden, relieving caller’s own suffering, struggling against external events, avoiding responsibility, etc.). For each question from (5) to (8), the caller was required to choose the most suitable one from the options of the question.

The demographic and socio-economic characteristics of the callers were also collected during the call. The variables included age, sex, education years, marital status and employment status, which has been deemed as important correlates of self-harm.

If callers did not know or answer any of the questions, the response was coded as *missing*.

The present study focused on characteristics of the most recent episode of non-fatal self harm reported by callers. There was no follow-up after the identified self-harm.

### Statistical analyses

The callers were divided into two groups, that is, treated self-harm callers (who received hospital-based medical treatment due to the most recent self-harm) and untreated self-harm callers (who did not receive such treatment due to the self-harm). Characteristics of the most recent episode of self-harm were compared between the treated and untreated self-harm callers, using chi-square test. Odds ratios (ORs) and 95% confidence intervals (CIs) of the correlates of untreated self-harm were calculated. A multivariate logistic regression model was used to explore the correlates of untreated self-harm callers. All socio-demographic variables were entered into the logistic regression model, and the other variables were selected using forward method. A further analysis was conducted by splitting the data by sex and reanalyzing the data of the male and female subsamples. Data analyses were run by SPSS 18.0. Two-tailed test and an alpha of 0.05 were used.

## Results

**Figure 1:**
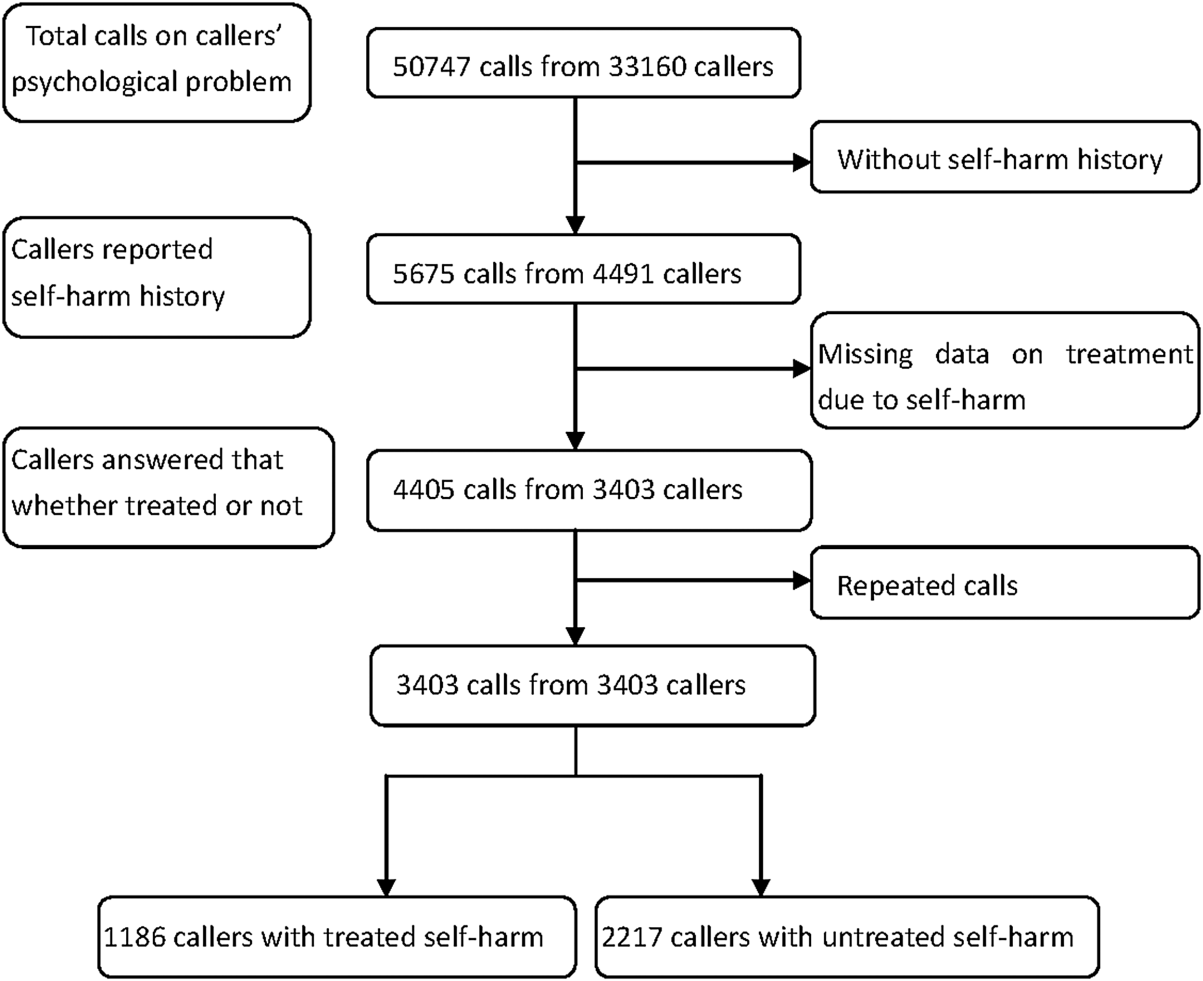
Flow chart of recruiting callers.

Figure 1 showed the recruiting process. From December 2002 to December 2008, 50,747 calls from 33,160 callers sought help due to psychological problems. Among those, 5,675 calls from 4,491 callers reported a history of self-harm. A total of 1,270 calls (1,088 callers) were further excluded because their information on medical treatment was missing. For the remained 3,403 callers, repeated calls were excluded due to duplicate information on the most recent self-harm episode. Finally, only one call from each of the 3,403 callers was included in the present study.

Compared with 1,088 excluded callers, the 3,403 included callers were less likely to be married (29.3% vs 33.1%, χ^2^ = 5.48, *p* = 0.019) and employed (45.3% vs. 51.2%, χ^2^ = 11.50, *p* = 0.001). They were slightly younger ((26.7 ± 8.5) years vs. (27.7 ± 8.5) years, *t* = −3.42, *p* = 0.001) and had more years of education ((12.1 ± 3.0) years vs. (12.5 ± 3.1) years, *t* = −3.56, *p* < 0.001). However, the proportion identifying as female (62.5% vs. 60.3%) and having the wish to die (62.0% vs. 61.0%) were not significantly different (*p* = 0.179 & 0.563, respectively) between included and excluded callers.

Among the 3,403 included callers, 1,571 (46.2%) reported two or more episodes of self-harm, and the others reported only one episode. The median (Inter-Quartile Range) time interval from the time of the most recent episode of self-harm to the index call was 360 (30–1,474) days. A total of 65% (2,217) of the included callers were untreated self-harm callers. Compared with treated self-harm callers, the untreated self-harm callers were younger and less likely to be married (see Table 1).

**Table 1:** Descriptive statistics for hotline callers with self-harm behavior.

Variables	All SH^a^ callers (*n* = 3,403)	Treated SH^b^ callers (*n* = 1,186)	Untreated SH^c^ callers (*n* = 2,217)	Comparison between treated and untreated SH callers	
	n (%)	n (%)	n (%)	χ^2^	*p*-value
Age (years)	26.7 (8.6)	28.2 (8.7)	25.9 (8.5)	7.23	<0.001
Education (years)	12.1 (3.1)	12.1 (3.1)	12.2 (3.1)	−0.39	0.697
	*n* (%)	*n* (%)	*n* (%)	χ^2^	
Female	2,128 (62.5)	748 (63.1)	1,380 (62.2)	0.223	0.637
Marital status				**42.674**	**<0.001**
Never married	2,149 (63.2)	668 (56.5)	1,481 (66.9)		
Currently married	997 (29.3)	395 (33.4)	602 (27.2)		
Divorced/separated	245 (7.2)	118 (10.0)	127 (5.7)		
Widowed	7 (0.2)	2 (0.2)	5 (0.2)		
Missing	5 (0.1)				
Work status				**49.441**	**<0.001**
Employed	1,539 (45.2)	554 (46.8)	985 (44.5)		
Student	974 (28.6)	260 (21.9)	714 (32.2)		
Unemployed	751 (22.1)	309 (26.1)	442 (20.0)		
Other	136 (4.0)	62 (5.2)	74 (3.3)		
Missing	3 (0.1)				
Wish to die				**118.596**	**<0.001**
Yes	2,106 (61.9)	880 (74.4)	1,226 (55.3)		
No	1,292 (38.0)	303 (25.6)	989 (44.7)		
Missing	5 (0.1)				
Methods of SH				**488.438**	**<0.001**
Medication overdose	927 (27.2)	536 (45.2)	391 (17.6)		
Other poisons	294 (8.6)	178 (15.0)	116 (5.2)		
With instrument^d^	1,424 (41.8)	344 (29.0)	1,080 (48.7)		
Jumping	115 (3.4)	32 (2.7)	83 (3.7)		
Other methods	641 (18.8)	95 (8.0)	546 (24.6)		
Missing	2 (0.1)				
Where SH occurred				3.665	0.160
Home	2,304 (67.7)	827 (69.7)	1,477 (66.6)		
Workplace	285 (8.4)	96 (8.1)	189 (8.5)		
Other public place	812 (23.9)	262 (22.1)	550 (24.8)		
Missing	2 (0.1)				
Main reason of SH				**29.302**	**<0.001**
Problems in romantic relationship	662 (19.5)	250 (21.1)	412 (18.6)		
Family conflict	908 (26.7)	342 (28.9)	566 (25.6)		
Conflict with persons outside family	314 (9.2)	90 (7.6)	224 (10.1)		
Work or study problems	324 (9.5)	81 (6.8)	243 (11.0)		
Poor or financial problem	36 (1.1)	12 (1.0)	24 (1.1)		
Physical illness	76 (2.2)	30 (2.5)	46 (2.1)		
Feeling depressed	805 (23.7)	270 (22.8)	535 (24.1)		
Other psychological problems	270 (7.9)	108 (9.1)	162 (7.3)		
Missing	8 (0.2)				
Main goal of SH				**23.259**	**0.002**
Decreasing others’ burden	108 (3.2)	43 (3.6)	65 (2.9)		
Relieving suffering	2,263 (66.5)	823 (69.6)	1,440 (65.0)		
Struggling against external events	253 (7.4)	78 (6.6)	175 (7.9)		
Avoiding responsibility	112 (3.3)	36 (3.0)	76 (3.4)		
Financial problems	132 (3.9)	53 (4.5)	79 (3.6)		
Punishing others	200 (5.9)	68 (5.7)	132 (6.0)		
Other	330 (9.7)	82 (6.9)	248 (11.2)		
Missing	5 (0.1)				

**Notes:**

a SH, self-harm.

b Treated SH callers, hotline callers who received medical treatment after self-harm.

c Untreated SH callers, hotline callers who did not receive medical treatment after self-harm.

d Using instrument refers to cutting by knives, hanging, or firearm.

Bold values indicate statistical significance.

As shown in Table 1, 55.3% of the 2,217 untreated self-harm callers reported a wish to die. However, callers reporting the wish to die by their most recent self-harm episode were less likely to be untreated (OR = 0.43, (95% CI [0.37–0.50]). Using instruments such as knife, etc. and medication overdose were common for all of the 3,403 included self-harm episodes. Callers who reported using medication overdose (OR = 3.85, (95% CI [3.29–4.52]) or ingestion of other poisons (OR = 3.20, (95% CI [2.50–4.09]) were more likely to be treated; however, those using instruments (OR = 0.43, (95% CI [0.37–0.50]) or other methods (OR = 0.27, (95% CI [0.21–0.34]) to self-harm were less likely to be treated.

Regarding wish to die, callers who had used overdosing on medication (χ^2^ = 104.18, *p* < 0.001), ingestion of other poisons (χ^2^ = 34.51, *p* < 0.001), or jumped (χ^2^ = 5.85, *p* = 0.016) were more likely to have the wish to die, and the callers using instruments (χ^2^ = 128.46, *p* < 0.001) or other methods (χ^2^ = 7.05, *p* = 0.008) were less likely to have the wish to die.

**Figure 2:**
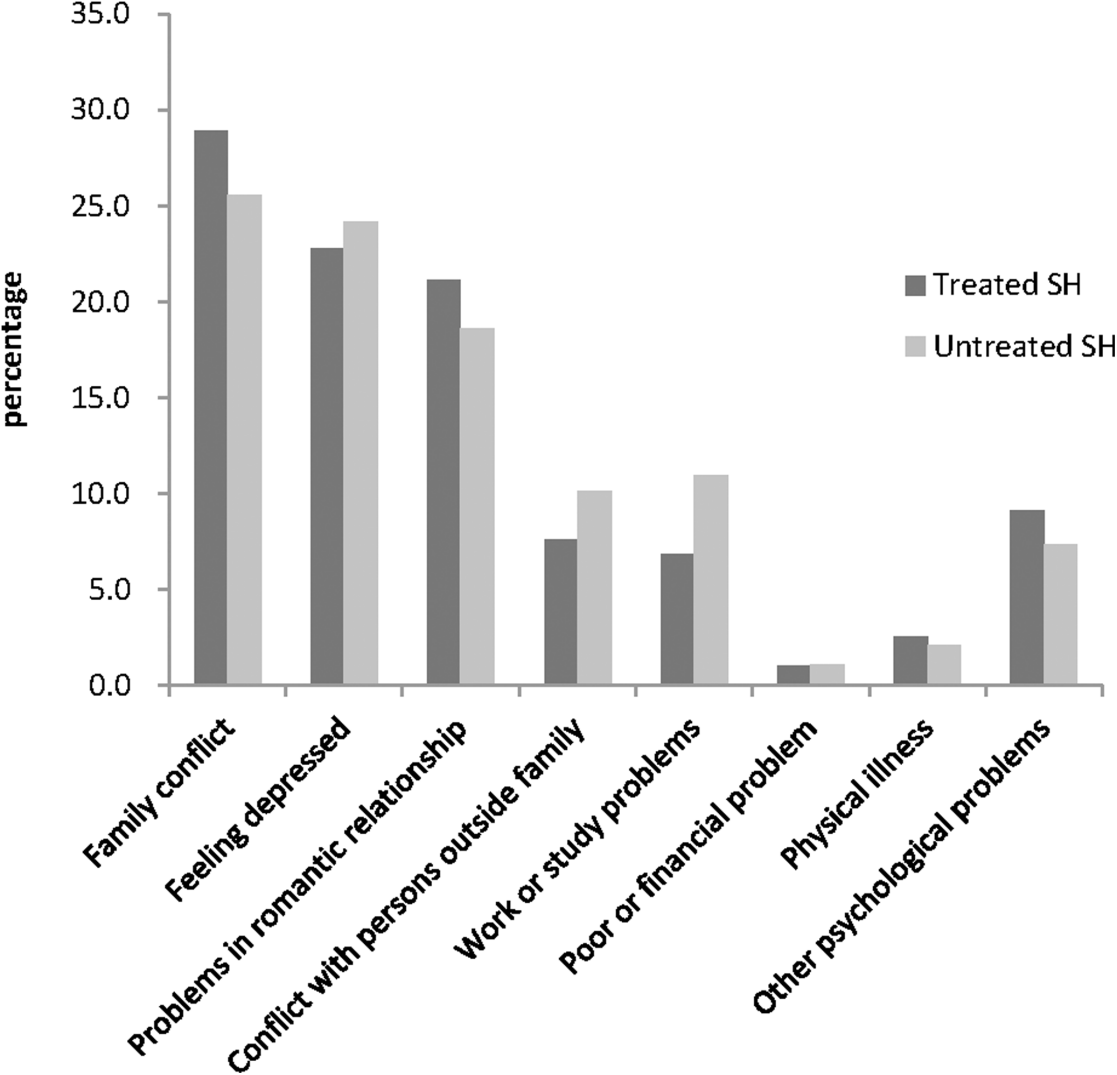
Percentages of different main reasons of self-harm.

Among all included self-harm episodes, 67.7% of self-harm occurred at home. Family conflicts, feeling depressed, and problems in romantic relationships were the most commonly reported reasons for self-harm (see Fig. 2). Treated self-harm callers were more likely to attribute the self-harm to family conflict (χ^2^ = 4.34, *p* = 0.037), and problems in romantic relationships with a marginal statistical significance (χ^2^ = 3.09, *p* = 0.079). Two-thirds of the callers reported relieving suffering as the main goal of self-harm behavior, and they were more likely to be treated (OR = 1.23, (95% CI [1.06–1.43]) and to have wish to die (OR = 3.29, (95% CI [2.84–3.82]).

Results in Table 2 indicate that, compared with treated self-harm callers, the untreated self-harm callers were less likely to report the wish to die at the time of most recent self-harm episode, to engage in self-harm at workplace or other public places, and to report problems in romantic relationships as the main reason for their self-harm episode, but they were more likely to use instruments (knife, etc.), jumping, or other methods for self-harm (AORs ranged from 3.73 to 7.71, see Table 2), after adjusted for demographic variables. While we divided the data into male and female subsamples, the results of the two subsamples indicated similar findings with combined sample, except for the place where self-harm occurred did not associated with receiving medical treatment in either of the male or the female subsample.

**Table 2:** Correlates of not receiving medical treatment (ref: treated in hospital) due to self-harm among hotline callers (*n* =3,348)^a^.

Variables	AOR^b^	95% CI^c^	*p*-value
Female	1.16	[0.98–1.38]	0.066
Age (years)	0.99	[0.98–1.01]	0.249
Education (years)	1.01	[0.98–1.04]	0.519
Marital status			
Never married	1.00		
Currently married	0.86	[0.68–1.09]	0.197
Divorced/separated	**0.63**	[0.45–0.89]	0.010
Widowed	3.64	[0.64–20.59]	0.144
Work status			
Student	1.00		
Employed	1.00	[0.78–1.29]	0.970
Unemployed	0.79	[0.61–1.03]	0.064
Other	0.85	[0.52–1.40]	0.482
Methods of self-harm			
Medication overdose	1.00		
Other poison	0.92	[0.70–1.21]	0.524
With instrument	**3.73**	[3.09–4.50]	<0.001
Jumping	**3.83**	[2.45–5.97]	<0.001
Other	**7.71**	[5.92–10.06]	<0.001
Wish to die	**0.57**	[0.48–0.67]	<0.001
Where self-harm occurred			
Home	1.00		
Workplace	**0.71**	[0.53–0.96]	0.024
Other	**0.78**	[0.64–0.96]	0.016
Main reason of self-harm			
Feeling depressed	1.00		
Problems in romantic relationship	**0.76**	[0.60–0.97]	0.027
Family conflict	0.85	[0.68–1.07]	0.176
Conflict with persons outside family	1.15	[0.84–1.57]	0.378
Work or study problems	1.29	[0.94–1.78]	0.109
Poor or financial problem	1.80	[0.79–4.08]	0.156
Physical illness	1.03	[0.61–1.76]	0.889
Other psychological problems	0.77	[0.56–1.05]	0.103

**Notes:**

a Fifty-five callers were excluded because of missing data.

b AOR, adjusted odds ratio.

c CI, confidence interval.

Bold values indicate statistical significance.

## Discussion

The characteristics of individuals who reported non-fatal self-harm behaviors and did not receive medical treatment in hospital due to the most recent self-harm episode have not been described well in the literature because of the difficulties in recruiting this sample. In the present study, we describe the characteristics of hotline callers and most recent episode of non-fatal self-harm. In general, among hotline callers, the most common method for non-fatal self-harm was using an instrument, such as cutting; more than half of the non-fatal self-harm occurred at home, and; more than half of the callers reported relieving suffering as the main goal. Notably, more than half of the self-harm callers were untreated, although 55% of the untreated callers reported the wish to die at the time of the most recent episode of self-harm. Compared with treated self-harm callers, the untreated self-harm callers were less likely to report the wish to die by self-harm, or engage in self-harm outside the home, and were more likely to use instruments as the means for self-harm.

Strikingly, substantial amounts of hotline callers who reported a history of non-fatal self-harm were untreated. Previous studies based on self-reports (O’Connor, Rasmussen & Hawton, 2012; Law & Shek, 2013; Liu et al., 2018; Tang et al., 2018) have reported a higher prevalence of self-harm behaviors than those based on hospital surveillance systems (Caterino et al., 2013; Haagsma et al., 2016; Tong et al., 2013a; Lyons et al., 2016). Many untreated self-harm incidences and the misclassification of self-harm in emergency rooms (Xu et al., 2013) might contribute to the huge gap between self-report and hospital-based surveillance systems. Social stigma related to suicidal behavior (Li et al., 2015) might be an important barrier to seeking treatment. These findings also suggest that using the hospital-based surveillance system may substantially underestimate the prevalence of non-fatal self-harm.

In addition, the method of non-fatal self-harm is an important predictor of seeking treatment. In previous hospital-surveillance-based studies in China (Tong et al., 2013a; Xu et al., 2013; An et al., 2014; Kwok et al., 2014) and other countries (Murphy et al., 2012), the most common method of self-harm with subsequent hospital-based treatment was self-poisoning (medication, pesticide, or other poisons). Our findings are consistent with the previous studies in that 60% of the treated self-harm callers have overdosed medications or ingested other poisons. However, self-harm callers using less medically damaged methods were less likely to seek for treatment. Self-harm callers using instruments, such as cutting, may be more easily treated outside of a medical system, such as dressing the wound themselves. The self-harm using other lethal methods, however, such as jumping, may have resulted in injuries very serious. This might account for why only a few callers who self-harmed by highly lethal methods were treated in the hospital.

Reporting the wish to die appeared to be another important predictor of receiving hospital-based medical treatment. A stronger wish to die by self-harm would result in using highly lethal method which needs medical treatment (Rivlin et al., 2012; Brown et al., 2014; Gjelsvik et al., 2016). The literature suggests that there is an incongruence between the wish to die and the self-harm method selected (Horesh, Levi & Apter, 2012). This may result from an inaccurate estimation made by individuals (Horesh, Levi & Apter, 2012; Brown et al., 2014). The wish to die is highly connected with psychological pain (Horesh, Levi & Apter, 2012; Sun & Zhang, 2016; Meerwijk & Weiss, 2017). In our study, relieving suffering was the most common goal of self-harm and was associated with the wish to die. Furthermore, the wish to die was also associated with medication overdosing or poison ingestion. It is possible that the callers with the wish to die were more likely to be treated in the hospital.

On the other hand, self-harm without the death intention or non-suicidal self-injury (NSSI) (Kapur et al., 2013; Klonsky, May & Glenn, 2013) may be similar enough to attempted suicide, and NSSI is also a strong predictor of suicide (Kapur et al., 2013; Klonsky, May & Glenn, 2013). In the present study, the wish to die distinguished untreated self-harm callers from the treated self-harm callers (OR = 0.57). Nevertheless, half of the individuals with prior untreated self-harm had the wish to die, and a quarter of the treated self-harm callers did not have the wish to die. Further research is needed to discriminate NSSI from attempted suicide with the wish to die.

Even after adjusting for demographic variables, other correlates remained statistically significant for whether being medically treated or not. Self-harm acts occurred at the workplace or other public places were more likely to be discovered by others and it was more possible for self-harm callers to be sent to hospitals for treatment. Compared with feeling depressed, the other two common reasons for self-harm, problems in romantic relationships or family conflict were correlated with seeking treatment (ORs < 1 in Table 2 means less likely to be untreated). However, only problems in romantic relationships reached statistical significance. It may be easier for interpersonal problems to be attended than depression, and it may make the self-harm behavior subsequent to interpersonal problems more likely to be discovered and then treated. Moreover, according to interpersonal theory, callers who faced interpersonal problems were more likely to have a desire of self-harm, and they tended to use methods which often need treatment, such as self-poisoning, to relieve interpersonal distress (Klonsky, May & Glenn, 2013).

There were some differences in our results on the characteristics of treated self-harm, compared with previous hospital surveillance data in China (Tong et al., 2013a; An et al., 2014). One study in Beijing found more than 70% of the self-harm patients treated in the emergency rooms overdosed medication (An et al., 2014). The other study in rural China revealed 67% of the self-harm treated in hospitals ingested pesticide (Tong et al., 2013a). However, our study found only 45% and 15% of the self-harm callers overdosed medications or used other poisons, respectively. The explanation might be that the recruited self-harm in the present study represented a broader population than previous studies did. In our study, self-harm with less severe injuries was included. These results highlight the importance of local resources to respond to and treat self-harm behaviors both in inpatient and outpatient settings. Moreover, treated self-harm callers were younger than those in the previous two studies and may suggest changing attitudes in awareness of suicide risk and access to treatment.

This study has several limitations. First, hotline callers were different from community residents or college students. The callers in our study were younger, well-educated, and more willing to seek psychological help via hotlines. Second, a quarter of samples (*N* = 1,088) were excluded because missing variables on prior treatment so the result was poor representative for all the callers. Third, all data were collected through self-report during telephone interviews and therefore the information had retrospective bias and social desirability bias. Fourth, the study did not differentiate the specific type of medical treatment, namely, outpatient, inpatient, or emergency room. Fifth, confounding factors, such as depression (Tong et al., 2013b; Yi & Hong, 2015), chronic physical health, disability and neighborhood characteristics, were not included in the analysis. Finally, we did not divide self-harm methods of using instruments into specific subgroups, such as self-cutting, fire-arm, or hanging, making it impossible to explore the association between the lethality of self-harm method and the medical treatment.

## Conclusions

This is the first study in China to explore associations between the characteristics of hotline callers reporting self-harm behaviors and the hospital-based medical treatment for the most recent episode of self-harm. Our findings suggest that the episodes of self-harm may be seriously underestimated by hospital surveillance system only. The burden of self-harm behavior may be more serious than we had previously estimated. Treatment within hospital settings may not be sufficient, given the low rates of medical treatment for self-harm. The untreated self-harm callers were less likely to have the wish to die and more likely to use instruments instead of poisons compared with treated self-harm callers. However, more than half of the untreated self-harm callers also had the wish to die. Public-health-focused suicide prevention strategies should involve individuals who have not received medical treatment for self-harm. It may be essential to provide effective self-harm intervention techniques outside and beyond traditional health care settings. Psychological hotline or internet-based mental health services (Lv et al., 2015) might be necessary for suicide prevention in China.

## Supplemental Information

10.7717/peerj.7868/supp-1 Supplemental Information 1 Variable labels and values in the study.

10.7717/peerj.7868/supp-2 Supplemental Information 2 Raw data.

## Additional Information and Declarations

### Data Availability

The following information was supplied regarding data availability: The data is available in the Supplemental Files.
