# Supplementary material for: Differences between medically treated and untreated non-fatal self-harm reported by hotline callers in China
Source: PeerJ. 2019 Oct 17;7:e7868. doi: 10.7717/peerj.7868 (PMC6800983; doi:10.7717/peerj.7868)
Supplement: Supplemental Information 1 [file peerj-07-7868-s001.docx]

Variables

codenum: case code

sex: 1=female, 2=male

age: age of caller

school: education years of caller

marital4: marital status of caller,1=never married, 2=current married, 3=divorce/separated, 4=widowed, 9=missing

wor4: employment status, 1=student, 2=unemployed, 3=wage earning job, 4=not paid, not working, 9=missing

suiatyn: reporting self-harm 1=yes, 2=no

prioratt.mult: reporting two or more episodes of self-harm, 1=yes, 0=no

datetime: the date of calling included in the study

suiatyy: the year of the most recent episode of self-harm

suiatmm: the month of the most recent episode of self-harm

suiatdd: the day of the most recent episode of self-harm

suiatloc: place where self-harm occurred, 1=home, 2=workplace, 3=public place, 8=other, 9=missing

suiatmet: self-harm methods, 1=poison, 2=medication, 3=instrument, 4=jumping, 8=other, 9=missing

suiatcau: main reason of self-harm. 1=love problem, 2=family conflict, 3=conflict with non-family persons, 4=work or study problems, 5=impoverished, 6=other money problems, 7=physical illness, 8=depression, 9=alcohol problem, 10=other psychological problem, 11=spiritual forces, 88=other, 99=missing

suiatobj: main goal of self-harm. 1=decrease others burden, 2=relieve suffering, 3=opposition to circumstances, 4=avoid responsibility, 5=financial problems, 6=punish others, 7=threaten others, 8=other, 9=missing

suiatdie: had a wish to die. 1=yes, 2=no, 3=unknown

suiatmed: whether treated in hospital (including inpatient and outpatient). 1=yes, 2=no, 3=unknown.
